# Intradialytic exercise in the treatment of social frailty: a single-center prospective study—preliminary results during the unexpected COVID-19 pandemic

**DOI:** 10.1186/s41100-020-00285-w

**Published:** 2020-08-05

**Authors:** Koki Abe, Yoshinosuke Shimamura, Takuto Maeda, Yoshikazu Kato, Yasuyoshi Yoshimura, Tomomi Tanaka, Hideki Takizawa

**Affiliations:** 1 Department of Nephrology, Teine Keijinkai Medical Center, 1-12 Maeda, Teine-ku, Sapporo, Hokkaido 006-8555 Japan; 2 Division of Rehabilitation, Teine Keijinkai Medical Center, Sapporo, Japan; 3 Division of Nutrition, Teine Keijinkai Medical Center, Sapporo, Japan

**Keywords:** Hemodialysis, Intradialytic exercise, Social frailty, Physical function, COVID-19

## Abstract

**Background:**

Social frailty—the lack of a connection to society and infrequent social activities—has been reported to be associated with future declines in physical function in elderly individuals. This study aimed to evaluate both the association of social frailty with the physical function and the efficacy of intradialytic exercise as a therapy for social frailty among hemodialysis patients.

**Methods:**

All 16 outpatient hemodialysis patients in the hemodialysis department of a single medical center were enrolled in this single-center prospective single-arm interventional study. Patients received five questions which asked about going out infrequently, lack of visiting friends, feeling unhelpful to friends or family, living alone, and lack of talking with someone. Those to whom two or more of the above were applicable were categorized as socially frail. All patients were placed into exercise therapy to be performed during their thrice-weekly hemodialysis visits. Participants’ physical function (walking speed), muscle strength (grip strength), muscle mass (appendicular skeletal muscle mass index), and social frailty were evaluated at baseline and after 3 months of therapy.

**Results:**

Four (25%) of the 16 participants (median age 71.5 years, 8 women) were categorized as being socially frail. In comparison to the non-socially frail group (non-SF), the socially frail group (SF) had a significantly lower walking speed (0.70 ± 0.12 m/s vs 1.15 ± 0.26 m/s, *p* = 0.005) and significantly worse performance on the Short Physical Performance Battery. Three months of intradialytic exercise therapy significantly improved their walking speed, from 1.04 ± 0.30 m/s to 1.16 ± 0.29 m/s (*p* = 0.003). intradialytic exercise therapy significantly improved walking speed in both the SF group and the non-SF group. The 2019 coronavirus disease pandemic unexpectedly occurred in the middle of the intervention period of this study, and although it was not statistically significant, the number of socially frail individuals among our participants increased to seven (43.8%, *p* = 0.248).

**Conclusions:**

Social frailty was associated with reduced physical function among hemodialysis patients. Intradialytic exercise therapy improved physical function regardless of the presence of social frailty.

**Trial registration:**

UMIN-CTR, UMIN-CTR000038313. Registered November 1, 2019, https://upload.umin.ac.jp/cgi-open-bin/ctr_e/ctr_view.cgi?recptno=R000043639.

## Background

Social frailty is the state of having reduced connections to society and little social activity. Among elderly community residents, social frailty has been reported to be significantly correlated with reduced physical function and future disability [1, 2]. As the coronavirus disease 2019 (COVID-19) pandemic continues to limit the social activity of people throughout the world, we must investigate how best to address social frailty.

Hemodialysis patients are also at high risk of losing physical function over time [3]. However, the prevalence of social frailty among dialysis patients, the effects of social frailty on their physical function, and effective therapies of social frailty remain unclear.

This study aimed to evaluate both the association of social frailty with the physical function and the efficacy of intradialytic exercise as a therapy for social frailty among hemodialysis patients.

## Methods

### Study design and intervention

This was a single-center prospective single-arm interventional study. All 16 patients undergoing outpatient hemodialysis at our hemodialysis center as of November 1, 2019 were selected for enrollment into this study. Exclusion criteria were as follows: cases for which we were unable to obtain consent and cases with severe cardiac illnesses (acute myocardial infarction, unstable angina, arrhythmia with hemodynamic abnormalities, and advanced aortic stenosis). All our 16 outpatient hemodialysis patients were interested in participating in our study and signed the written, informed consent form. None of the 16 participants met any of these exclusion criteria.

The intervention in this study was thrice-weekly intradialytic (i.e., during hemodialysis) exercise therapy. Patients underwent 50 min of exercise therapy during each of their three-weekly hemodialysis visits. Exercise therapy began 1 h after dialysis therapy had begun, and consisted of 10 min of stretching, 20 min of aerobic exercise using an ergometer at a rating of perceived exertion (RPE) of 13 (“somewhat hard”), and 20 min of resistance training using elastic bands and weights at an RPE of 13. Resistance training consisted of shoulder flexion/abduction, hip flexion/abduction, knee extension, and back bridge exercises, with 20 repetitions constituting one set of each exercise. Dedicated physical therapists were present to monitor exercise sessions and offer advice. This study was registered with the Japanese University Hospital Medical Information Network Clinical Trials Registry (UMIN 000038313). The study protocol used herein was approved by the Internal Review Board of the Teine Keijinkai Medical Center (IRB Approval No. 2-019071-00) and was carried out in accordance with the Declaration of Helsinki.

### Data collection

We recorded information on patients’ demographic characteristics, history of dialysis, comorbidities, number of medications currently being taken, social frailty status, physical function, muscle strength, muscle mass, and laboratory data.

### Social frailty

In keeping with the approach taking by Makizako et al., we evaluated patients’ social frailty status by asking five questions [1]. These were as follows: (1) Do you go out less often now than you did a year ago? (2) Do you rarely visit the homes of your friends? (3) Do you feel that you are unhelpful to your family and friends? (4) Do you live alone? (5) Do you not have the opportunity to speak to someone every day? Patients that answered “Yes” to two or more of these questions were categorized as socially frail.

### Measurement of outcome

Patients were measured at baseline and after 3 months of therapy. Measurements of physical function were carried out on dialysis therapy days before dialysis. Outcomes of this study were improvements in patients’ walking speed, physical function, muscle strength, muscle mass, and social frailty status after 3 months of treatment.

### Physical function and muscle strength

Physical function was evaluated via walking speed and the Short Physical Performance Battery (SPPB). Walking speed was measured as patients’ normal walking speed, without speeding up or slowing down, over a distance of 4 m. Speeds of 0.8 m/s or lower were defined as reduced walking speed [3]. The SPPB was carried out in accordance with the standard method: patients were given scores from 0–4 based on their performance in each of three tasks: balance test, gait speed test, chair stand test. These three scores were totaled to yield a composite score [4]. Muscle strength was evaluated as grip strength, which was measured while the patient was standing with their arms extended. Left and right arms were each measured twice, and the highest value was recorded.

### Muscle mass

Muscle mass was evaluated using the appendicular skeletal muscle mass index (ASMI). First, patient muscle mass was recorded using bioelectrical impedance analysis (BIA), which was then divided by the patient’s height in meters and squared to yield an ASMI value.

### Statistical analysis

Normal distribution was assessed by the Shapiro-Wilk test. Continuous variables are expressed as means and standard deviation (SD) or median and interquartile range (IQR 25–75th percentile) depending on the results of the Shapiro-Wilk test. Categorical variables were expressed as frequency and percentage. Differences in continuous variables between patients with social frailty and those without were tested by Student’s *t* test or the Mann–Whitney *U* test. Differences in continuous variables within a group were tested by paired *t* test or the Wilcoxon signed-rank test. Differences in categorical variables between patients with social frailty and patients without were analyzed by Fisher’s test. Differences in categorical variables within a group were tested by McNemar test. Spearman’s rank correlation was used to determine the relationship between the gait speed and the social frailty score. A probability value of < 0.05 was considered to be statistically significant. Statistical analysis was performed using the EZR software (Jichi Medical University, Saitama, Japan).

## Results

**Fig. 1:**
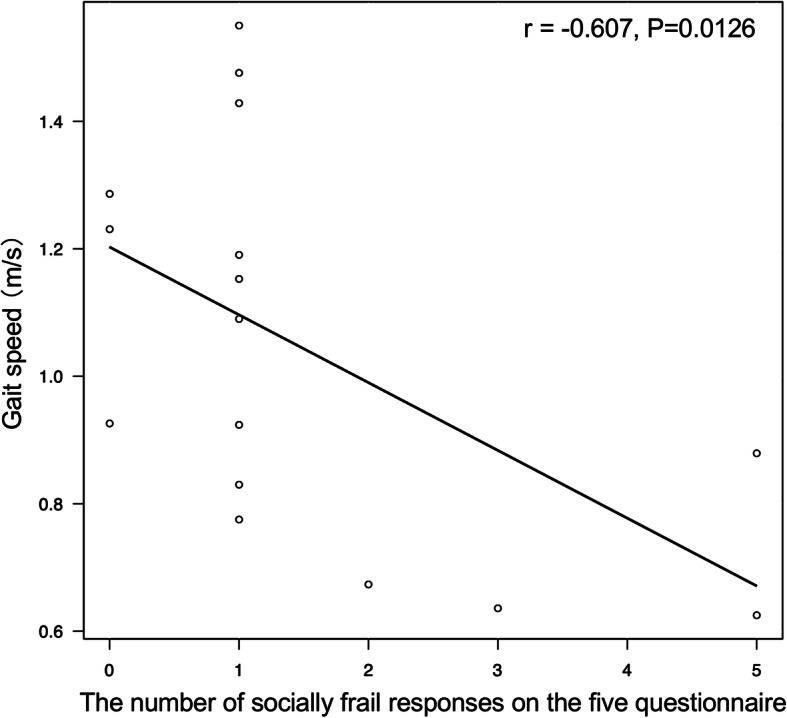
Correlation between social frailty score and gait speed

Patients’ background characteristics by social frailty status are given in Table 1. Four (25%) of the 16 participants in this study (median age 71.5 years, eight women) were deemed to be socially frail. In comparison to the non-socially frail group (non-SF), the SF group had a significantly slower walking speed (0.70 ± 0.12 m/s vs 1.15 ± 0.26 m/s, *p* = 0.005) and significantly worse performance on the SPPB. The number of socially frail responses on the SF questionnaire correlated negatively with walking speed (*r* = − 0.607, *p* = 0.012, Fig. 1) and the SPPB (*r* = − 0.653, *p* = 0.006). Single regression analysis indicated that social frailty was a risk factor for reduced walking speed (odds ratio 33.0, 95% confidence interval 1.56–698.00, *p* = 0.025).

**Table 1:** Baseline characteristics of study subjects

Characteristics	Total	Non-social frailty	Social frailty	
	(*n* = 16)	(*n* = 12)	(*n* = 4)	*P* value
Age (years)	71.5 (64.8–78.0)	70.5 (64.8–73.5)	76.0 (69.5–78.3)	0.43
Female sex (%)	8 (50.0)	6 (50.0)	2 (50.0)	1.0
Height (cm)	159.6 ± 10.4	161.2 ± 8.6	154.6 ± 14.9	0.28
Body weight (kg)	51.4 ± 12.2	52.1 ± 12.7	49.2 ± 12.1	0.69
Body mass index (kg/m^2^)	19.5 (18.5–20.7)	19.1 (17.8–20.7)	20.3 (20.0–20.7)	0.47
Dialysis vintage (months)	83.1 ± 60.4	68.4 ± 49.0	127.3 ± 77.3	0.092
Charlson comorbidity index	5.0 (4–6.3)	5.0 (4.8–6.3)	4.0 (4.0–4.8)	0.23
Number of medications	6.3 ± 3.0	6.2 ± 3.4	6.8 ± 1.5	0.75
Gait speed (m/sec)	1.04 ± 0.30	1.15 ± 0.26	0.70 ± 0.12	0.005
SPPB	12.0 (10.0–12.0)	12.0 (12.0–12.0)	10.0 (9.5–10.3)	0.008
Grip strength (kg)	25.1 ± 7.7	25.8 ± 6.9	22.9 ± 10.6	0.53
ASMI (kg/m^2^)	6.4 ± 0.9	6.6 ± 0.9	6.1 ± 1.1	0.46
Hemoglobin (g/dL)	11.3 (11.1–11.7)	11.3 (11.3–11.7)	10.7 (10.0–11.2)	0.11
Albumin (g/dL)	3.65 ± 0.26	3.67 ± 0.21	3.58 ± 0.39	0.52
Blood urea nitrogen (mg/dL)	52.2 ± 12.9	54.5 ± 13.3	45.2 ± 9.8	0.22
Creatinine (mg/dL)	8.65 ± 2.22	8.58 ± 2.33	8.86 ± 2.16	0.84
Phosphate (mg/dL)	5.1 ± 1.4	5.0 ± 1.2	5.5 ± 2.0	0.53
Total cholesterol (mg/dL)	196.7 ± 41.3	196.6 ± 48.2	197.0 ± 5.0	0.99
C-reactive protein (mg/dL)	0.07 (0.04–0.11)	0.07 (0.05–0.09)	0.10 (0.04–0.30)	0.54

All measurements are represented as number (%), mean ± standard deviation, or median (interquartile range).

*SPPB* short physical performance battery, *ASMI* appendicular skeletal muscle mass

**Fig. 2:**
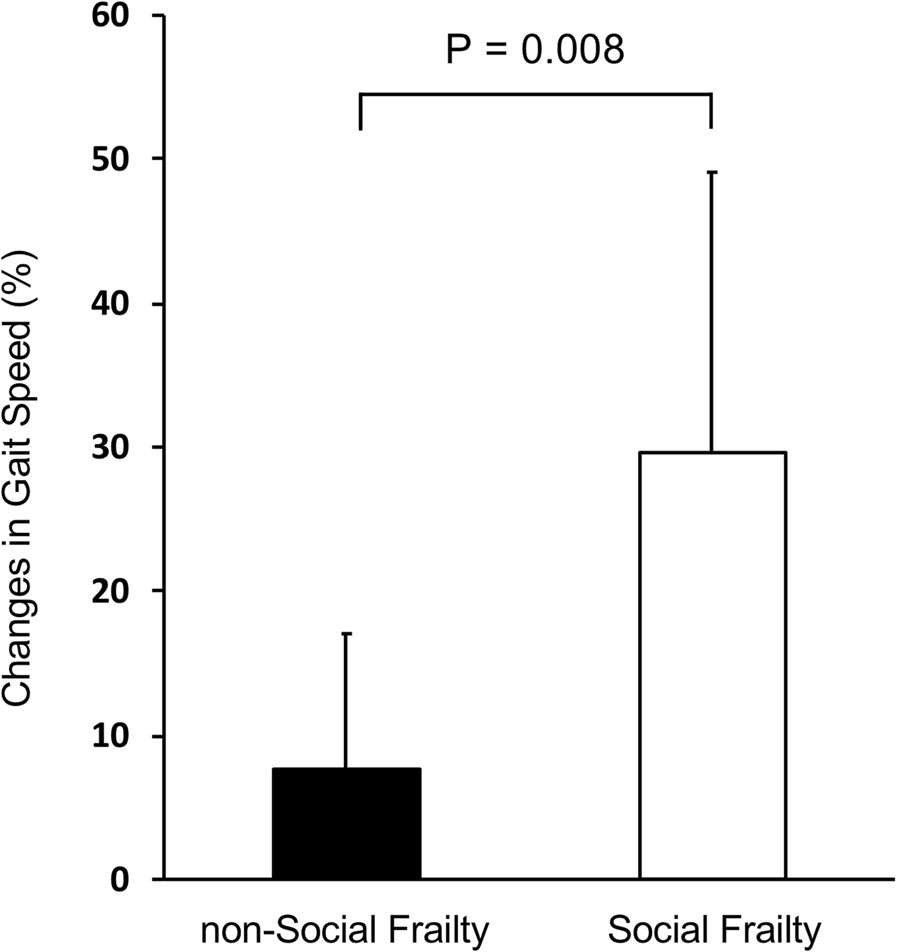
Percent changes in gait speed. Data are shown as mean and standard deviation

The outcomes of physical function, muscle strength, and muscle mass are listed in Table 2. Three months of intradialytic exercise therapy improved walking speed from 1.04 ± 0.30 m/s to 1.16 ± 0.29 m/s (*p* = 0.003); however, no significant change in SPPB performance was observed. ASMI, grip strength, serum albumin, and C-reactive protein likewise did not change. A significant improvement in walking speed was seen in both the SF and non-SF groups. Additionally, the improvement was larger in the SF group (Fig. 2).

**Table 2:** Changes in physical function, muscle strength, muscle mass, laboratory data, and social frailty

	Baseline	3 months	*P* value
**Total (*n*= 16)**			
Gait speed (m/sec)	1.04 ± 0.30	1.16 ± 0.29	0.003
SPPB	12.0 (10.0–12.0)	12.0 (12.0–12.0)	0.17
Grip strength (kg)	25.1 ± 7.7	24.5 ± 7.4	0.39
ASMI (kg/m^2^)	6.4 ± 0.9	6.4 ± 1.0	0.64
Albumin (g/dL)	3.65 ± 0.26	3.65 ± 0.22	1
C-reactive protein (mg/dL)	0.07 (0.04–0.11)	0.08 (0.05–0.12)	0.208
The number of socially frail responses	1.0 (1.0–1.3)	1.0 (1.0–3.0)	0.071
**non-social frailty (*n*= 12)**			
Gait speed (m/sec)	1.15 ± 0.26	1.24 ± 0.27	0.028
**Social frailty (*n*= 4)**			
Gait speed (m/sec)	0.70 ± 0.12	0.91 ± 0.20	0.0497

All measurements are represented as mean ± standard deviation or median (interquartile range).

*SPPB* short physical performance battery, *ASMI* appendicular skeletal muscle mass

Changes in indices of social frailty are given in Table 3. The number of individuals deemed socially frail increased from four to seven. Despite the fact that this increase was not statistically significant, a trending increase in the number of socially frail responses to our questionnaire was observed (*p* = 0.07, Table 2). During our research period, the question item whose responses showed the worst result was the frequency of going out.

**Table 3:** Changes in subitems of social frailty

	Total (*n* = 16)	
	Baseline	3 months
Going out infrequently (%)	8 (50)	11 (69)
Lack of visiting friends (%)	5 (31)	6 (38)
Feeling unhelpful to friends or family (%)	5 (31)	5 (31)
Living alone (%)	4 (25)	5 (31)
Lack of talking with someone (%)	2 (13)	3 (19)
Social frailty (%)	4 (25)	7 (44)

All measurements are represented as number (%)

No complications arose as a result of the intradialytic exercise therapy performed in this study. Further, no patients developed COVID-19 during the interventional period of this study, and no patients needed to suspend their dialysis therapy regimen.

## Discussion

### Summary of results

In this study, we evaluated the association of social frailty with the physical function and the efficacy of intradialytic exercise therapy in hemodialysis patients. Our results showed that social frailty was significantly correlated with decreased physical function and intradialytic exercise therapy that improved patients’ walking speeds regardless of their social frailty status.

### Most novel finding

We have demonstrated that social frailty was significantly correlated with decreased physical function among hemodialysis outpatients. The prevalence of social frailty observed in this study was higher than that reported among the general elderly population. A retrospective cohort study of elderly community residents reported a social frailty prevalence of 10.2% [1]. A prior study demonstrated that social frailty was associated with a loss of physical functionality and disability in community-dwelling elderly population [2]. Our data suggested that social frailty was also correlated with decreased physical function in hemodialysis patients.

### Potential mechanism

Our result can be explained by the fact that hemodialysis patients are prone to have sedentary lifestyles and to have low levels of physical activity [5, 6]. Other studies also reported that decreased physical activity was associated with reduced walking speed in hemodialysis patients [7, 8]. We can speculate that social frailty, such as a reduced frequency of going out and social visits with friends, reduces walking speed by limiting patients’ opportunities to engage in physical activity.

### Secondary finding

We showed intradialytic exercise therapy contributed to improving patients’ walking speeds both in the SF group and the non-SF group. Both intradialytic and interdialytic exercises have been shown to be effective [9]. We selected intradialytic exercise because intradialytic cycling and resistance training with guidance were shown to be effective in a short period [10, 11]. In contrast, interdialytic exercise, including home exercise therapy, had poor adherence [12, 13].

### Implications of the study

During our study, the first Japanese case of COVID-19 was reported on January 15, 2020. As of April 30, the Japanese government has intermittently requested the public to refrain from leaving their homes [14]. In our hemodialysis center, in order to prevent the spread of COVID-19, dialysis staff, physical therapists, and patients were all subjected to daily morning measurements of body temperature, asked to wear masks, requested to follow cough etiquette, and to practice proper handwashing. Staff that developed fever-like symptoms were instructed to stay at home until the possibility of COVID-19 infection could be ruled out. Patients that developed fever-like symptoms had their dialysis treatments adjusted so as to be separated from other patients, received dialysis therapy in a private room, and had their intradialytic exercise therapy postponed [15, 16]. The COVID-19 pandemic, which occurred in the middle of this study, has impacted on our patients because three new cases of social frailty arose. Thus, the number of dialysis patients with social frailty is expected to be increased during the COVID-19 pandemic, suggesting the need for care and monitoring to avoid further declines in physical function among this vulnerable population.

## Limitations

This study was subject to several limitations. First, it was a single-center study, meaning that some bias in patient selection may be present. Second, because its sample size is small, the effect of social frailty on the physical function could not be adequately adjusted for confounding factors like age, comorbidities, nutritional status, and cognitive function. A multicenter, large-scale trial is therefore necessary. Third, our study period was relatively short, so that long-term outcomes were not evaluated.

## Conclusions

We conducted a single-center interventional trial of intradialytic exercise therapy in hemodialysis patients. Our results suggest that social frailty is associated with reduced physical function and that, irrespective of social frailty status, intradialytic exercise therapy improved patients’ physical function. Fortunately, none of our patients developed COVID-19 during the interventional period of this study, but it is crucial to investigate the effect of the COVID-19 pandemic on physical function and intradialytic exercise therapy in our patients in our future research.

## Data Availability

The datasets used and/or analyzed during the current study are available from the corresponding author on reasonable request.

## Abbreviations

ASMI: Appendicular skeletal muscle mass index
BIA: Bioelectrical impedance analysis
COVID-19: Coronavirus disease 2019
IQR: Interquartile range
non-SF: Non-social frailty
RPE: Rating of perceived exertion
SD: Standard deviation
SF: Social frailty
SPPB: Short physical performance battery
